# School Nurses’ Perceptions and Experiences of Delivering a School-Based Intervention to Improve Primary Prevention of Human Papillomavirus Among Adolescents—A Focus Group Study Following a Randomized Controlled Trial

**DOI:** 10.1177/10598405211046174

**Published:** 2021-10-09

**Authors:** Magdalena Mattebo, Maria Gottvall, Maria Grandahl

**Affiliations:** 1 School of Health, Care and Social Welfare, Mälardalen University, Västerås, Sweden; 2 Department of Women's and Children's Health, 8097 Uppsala University, Uppsala, Sweden; 3 Department of Health Sciences, 7666 The Red Cross University College, Huddinge, Sweden

**Keywords:** family life/sexuality, health education, high school, school nurse, knowledge/perceptions/self-efficacy, qualitative research, immunizations

## Abstract

The present qualitative study is a part of the process evaluation of a complex intervention, the randomized controlled trial, “Prevention of human papillomavirus (HPV) in a school-based setting.” We aimed to explore participating school nurses’ perceptions and experiences of delivering the educational HPV intervention to adolescents aged 16. Focus group interviews were conducted with school nurses (*n* = 20) and analyzed with inductive qualitative content analysis. The overall theme Easily adapted into the existing role as a school nurse permeated the participants’ views. The nurses were in favor of delivering an intervention that increased the HPV vaccination rates and improved beliefs and awareness about HPV prevention. It suits their work and health-promoting aspect of their role well and can easily be adapted into the current school health consultant curriculum. Having material in different languages to share with adolescents and their parents to promote equal health was deemed important.

## Background

Human papillomavirus (HPV) is the most common sexually transmitted infection (STI) among both women and men in the world, and it is associated with cancer of the oropharynx, cervix, vagina, penis, anus, and vulva (Bray et al., 2018; Forman et al., 2012). Vaccination against HPV is effective and safe and has been included in the Swedish national vaccination program since 2012. This vaccination is offered free of charge to all girls aged 10–12 through the school healthcare system but requires parental consent. From 2020, boys have also been included in the national HPV vaccination program. Even if a majority of parents accept HPV vaccination for their child, the national coverage is lower (80%) for this vaccination compared to other vaccinations (97%) in the Swedish national vaccination program (Public Health Agency of Sweden, 2017). So far very few randomized trials have been conducted among adolescents with the aim to promote HPV prevention (Gottvall et al., 2010; Grandahl et al., 2019; Marek et al., 2012). Health care staff have an important role related to communication and providing information about the HPV vaccination (Boyce & Holmes, 2012; Gilkey et al., 2016; Rosen et al., 2016, 2019). Specifically, in Sweden, school nurses have a key role in this area since they are responsible for the vaccinations within the national vaccination program (Grandahl et al., 2017) and also for health consultations offered to all students on four occasions during the school years. At age 16, sexual health is included in the health consultation, and students complete a questionnaire about perceived health.

According to the Convention on the Rights of the Child (UNICEF, 2015), which became law in Sweden (2018:1197) in 2020, the best interests of the child should always be considered, and children have the right to be involved in all decisions regarding their health. Keeping this in mind, it is of significant importance that communication regarding HPV prevention is conducted in a way to improve knowledge among adolescents and their parents about why HPV prevention is important. This may increase vaccination uptake and condom use among adolescents. Previous studies indicate that interventions may improve children's vaccination status, parents’ knowledge, and parents’ intention to vaccinate (Fu et al., 2014; Kaufman et al., 2013). Interventions directed to parents and adolescents can also have a beneficial effect on beliefs about the HPV vaccination (Fu et al., 2014; Gottvall et al., 2010; Marek et al., 2012). As far as we know, no intervention aiming to increase HPV vaccination rates has been developed together with the stakeholders. Thus, health care professionals and school nurses, parents, and not to forget, children, should be engaged in this process. This is a way to strengthen children's voices in line with the Convention on the Rights of the Child (UNICEF, 2015) and is essential in the global battle to eliminate HPV-related cancer.

It is encouraging that boys are now included in the national vaccination program. However, knowledge of the virus and the HPV vaccine is low among adolescents and their parents, as well as in the population at large; in addition, few people are aware of the clear benefits of the HPV vaccination for boys (Gottvall et al., 2017; Perez et al., 2015). Moreover, vaccine hesitancy in society is a growing challenge (MacDonald & Hesitancy, 2015); in 2019, it was listed by the World Health Organization (WHO) as one of the top ten global public health threats. The stated WHO goal is to achieve a worldwide HPV vaccine coverage of ≥90%. Hence, more work is needed to achieve herd immunity and effective cancer prevention. The aim of the study was to explore school nurses’ perceptions and experiences of delivering a complex HPV intervention to adolescents.

## Method

### Design and Setting

The present qualitative interview study is a part of the process evaluation of a complex intervention, the randomized controlled trial (RCT), “Prevention of HPV in a school-based setting,” undertaken in Sweden. The study follows Standards for Reporting Qualitative Research (O'Brien et al., 2014) and is reported according to the COnsolidated criteria for REporting Qualitative research (COREQ) Checklist (Supplemental Material; Tong et al., 2007). A school-based cluster RCT intervention was conducted among 751 secondary school students aged 16 years (Grandahl et al., 2016). The overall aim was to improve primary prevention of HPV infection by promoting vaccination and increased condom use. The included schools were situated in city and rural areas, and the sample included both boys and girls. Both university preparatory and vocational programs were represented. The educational intervention included the school nurse providing 1) 30 min of face-to-face structured information about HPV and 2) a specially designed information leaflet to each student. The information covered the following components: facts about HPV, the link between HPV and cancer, and HPV prevention, (i.e., condom use), and HPV vaccination. Students in both the intervention group and the control group answered the questionnaires at baseline and after three months. Students in the control group received standard treatment, (i.e., the regular health consultation). The RCT showed favorable effects on the adolescents’ awareness and beliefs regarding HPV prevention. At follow-up after three months, more girls in the intervention group were vaccinated than among the controls (Grandahl et al., 2016). The Swedish Ethical Review Authority (D.nr. 2013/324) approved this project.

### Sample and Procedure

School nurses who had delivered the educational intervention about the HPV prevention described above were eligible to participate. Hence, nurses (*n* = 20), both in the intervention group (*n* = 10) and in the control group (*n* = 10), were invited to participate in focus group interviews.

Three focus group interviews were conducted in 2015–2016. Due to logistic reasons, one school nurse was interviewed by phone. All interviews lasted between 60–90 min and were audio-recorded. Each interview started with brief information about the study. All participants had given their written consent to participate in the study. The participants were informed that participation was voluntary, that they could withdraw at any time, for any or no given reason, without incurring any negative consequences for themselves. They were also informed that only the researchers would have access to the data and that all data would be presented on a group level. The interviews were transcribed verbatim, and no repeat interviews were carried out. All interviews were carried out by health professionals with a Ph.D. (MGr and a research colleague at Uppsala University), with documented experience of qualitative methods and the topic in question.

### Interview Guide

We used a semi-structured interview guide, based on our previous intervention study about reproductive health (Stern et al., 2015) and the questionnaire that is part of this project (Grandahl et al., 2016). In summary, the questions focused on the informant's perceptions and experiences of delivering the school-based intervention to adolescents, with a special emphasis on the vaccination for HPV and its prevention (Table 1).

**Table 1.** Interview Guide.

*Initial information: Short presentation by the interviewer about the human papillomavirus (HPV) project, “Prevention of HPV in a school-based setting,” the aim of the present focus group interview, and a short presentation of the present participants.*
Overall experiences of participation - What is your experience of participating in the HPV project?
Specific experiences of participation - What are your thoughts of perceived benefits (positive)? - What are your thoughts of perceived barriers (negative)?
Overall perceptions and thoughts of the concept “Prevention of HPV in a school-based setting” - What are your thoughts of providing information about HPV to students at the time for the general health consultation? - What are your thoughts of providing information about HPV to both girls and boys
Future aspects - What are your thoughts regarding continuing to inform about HPV at the time for the general health consultation? For your own part? To prevent illness among the students? What are your thoughts regarding including information about HPV at the time for the general health consultation as a standard information (routinely)? What are your thoughts of providing information about HPV to adolescents in other ways?—By whom?—How? Please describe.
To conclude - Is there anything more you would like to say which I didn’t ask you about?
The interviewer summarizes the interview. Thank you!

### Analysis

**Figure 1.**
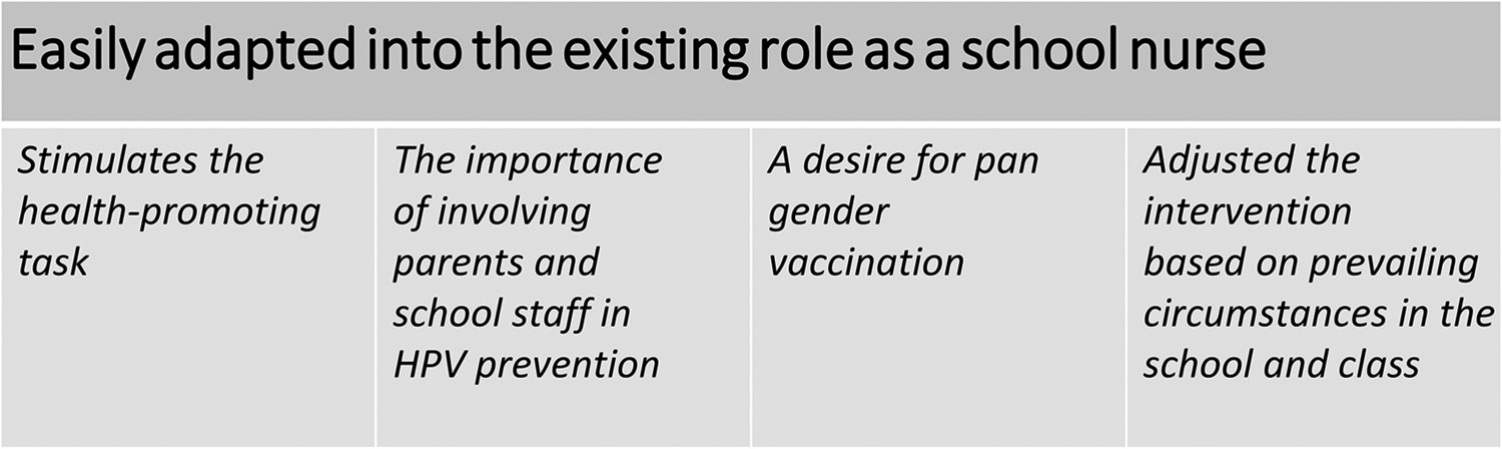
Theme and categories identified from the analysis.

We used qualitative content analysis with an inductive approach. The transcripts were read repeatedly to get an overall picture of the data. Units of meaning were extracted, condensed, and labeled with a color mark. These units were then sorted into categories by two researchers (MGo & MM) working individually. Finally, all the authors discussed the categories until a consensus was reached (Burnard et al., 2008; Lindgren et al., 2020). The analytic process went forward and back. NVivo software was used for managing the data. One theme and four categories were identified (Figure 1).

## Results

In total, 20 school nurses from secondary schools participated in this study. The age of the participants varied between 39 and 62. All participants were female. All except one had a specialist nurse degree (i.e., master's level). The schools represented both public and private schools and were situated in different geographic and socioeconomic areas in Sweden. The schools offered vocational as well as theoretical education.

One theme and four categories were determined: (1) The intervention *Stimulated the health-promoting task,* (2) emphasized *The importance of involving parents and school staff in HPV prevention*, (3) the school nurses *Expressed a wish for pan gender HPV vaccination*, (4) and they *Adjusted the intervention based on prevailing circumstances in the school and class*. Together, these make up the overall theme: *Easily adapted into the existing role as a school nurse*.

### Easily Adapted Into the Existing Role As a School Nurse

The overall theme that permeated the participants’ perceptions and experiences was that the intervention was easily adapted into their existing role as a school nurses. Health promotion is a part of everyday work as a school nurse, and even though HPV-related information is still not a part of the regular health consultations, this was viewed as an easy way of including information about HPV and HPV vaccination.

### Stimulates the Health-Promoting Task

Overall, the participants thought that the intervention stimulated the health-promoting aspects of their work. They pointed out that it enriched the current health-promoting visits to which the16-year-old students were invited. Furthermore, the intervention increased knowledge about HPV and STIs generally, both among the youth as well as among themselves. It was also seen as an opportunity to discuss sexual health with the adolescents. The leaflet helped to promote the practical advice regarding prevention against HPV. Informing adolescents about HPV had become a routine during health visits. However, the participants found it difficult to find time to talk about HPV within the current health visit, leading to feelings of stress and discomfort.

“*You are supposed to work health promotive regardless and include everything you can, try to include everything.”* (Focus group 3)

“*Well, they thought like ‘this does not apply to me.’ But then I could say, ‘if you are planning to have sex with anyone at any time in your life, that is enough; that is a reason to take the vaccine. And then, they could accept it.’”* (Focus group 2)

“*You feel a little stressed about time when you sit there and have scheduled appointments with all your students. And you can’t reschedule them all because then there is no chance whatsoever that you will have the time to do all the health visits*.” (Focus group 3)

### The Importance of Involving Parents and School Staff in HPV Prevention

The participants claimed that linking parents, adolescents, and school staff is a necessity in HPV prevention, although they would like to have more involvement in reaching parents. Another important aspect of involving parents and school staff is making sure the information and relevant material are norm conscious to contribute to the understanding that this applies to everybody, regardless of gender, sexuality, ethnicity, or any other sociodemographic factors.

“*Material that is adapted for parents. If I could have a parent evening. I think that would be great in the future, to ensure that even more students will be vaccinated when they get to secondary school and maybe you know more why you are being vaccinated and what it's for.”* (Focus group 4)

*“And same thing when it comes to STIs, that you understand, because the teacher could say, ‘ok, if you have more thoughts, anyone have questions about STIs. The school nurse, go and talk to her or talk about it during your health visit.’ That will be a great start.”* (Focus group 1)

### A Desire for Pan Gender HPV Vaccination

The participants expressed a vision about pan gender HPV vaccination. This was in regard to age, maturity, gender, disabilities, and ethnic background. Generally, it was more difficult to get boys interested as boys thought this had nothing to do with them, given that the aim of the vaccination is presented as a protection against cervical cancer. At the time of the interviews, the HPV vaccination was only offered to girls which made it even harder to get the boys interested.

If the intervention was to become permanent, it was considered a necessity to have material that could be adapted. The participants adjusted the health visit according to age and maturity. It was also of utmost importance to have written information in different languages to facilitate visits with youths coming from other countries and cultures.

“*You have to start early, but keep them updated throughout the school system, so that it will, because as I said you mature at different ages, you are receptive to different things.”* (Focus group 2)

“*Then, I have to say it's a shame that we didn’t start with the boys as well, at the same time as when we started with the girls. I can’t understand what they were thinking.”* (Focus group 3)

### Adjusted the Intervention Based on Prevailing Circumstances in School and Class

The participants adjusted their way of working with the intervention, based on circumstances in the school where they worked, depending on the specific class and students they met. They expressed having high ambitions in their work and pointed out a personal responsibility as a driver for achieving high quality. They had an ambition to see each person as an individual as well as his or her unique needs for information and counseling. In order to achieve this, they expressed a wish to know what the student desired when meeting the school nurse, to be able to adjust their work accordingly.

“*I dońt work like other nurses; I ask like this: ‘Between one and ten, how are you?’ If the answer is 10, then I say, ‘ok, you can go because then everything is fine.’ And they get very surprised and then we always talk about something. But if is seven or eight, I ask, ‘why do you say three points below and then we can address the problem immediately.’”* (Focus group 2)

“*The only thing I feel, with the girls who were there, I would have wanted to give them the vaccination when they were there. Because I think almost no one is going to go to the health care center and get it.”* (Focus group 4)

## Discussion

We interviewed school nurses who had delivered an educational intervention with the aim to improve primary prevention of HPV infection by promoting vaccination and increased condom use among high school students. The overall theme that permeated the participants’ perceptions and experiences was that the intervention was “Easily adapted into the existing role as a school nurse.” The participants thought that the intervention stimulated them to perform the health-promoting tasks, in accordance with their role as a school nurses. They also expressed a necessity of involving parents and school staff in HPV prevention and a vision of pan gender HPV vaccination.

According to the role held by school nurses, the participants emphasized the importance of health promotion among youth. HPV-related cervical cancer still results in over 300,000 deaths annually worldwide. Furthermore, an increase in HPV-associated oropharyngeal cancer among both women and men underlines the importance of early and repetitive health promotion among adolescents (Van Dyne et al., 2018; Zamani et al., 2020). The participants also stated that informing and educating adolescents about HPV along with the health-promoting task was in line with their role as a school nurse. However, they pointed out that it would not be possible to perform this task alone and having a close cooperation with principals and teachers was vital in succeeding with this task. This emphasizes the importance of giving school nurses the right conditions for them to be able to fulfill their tasks (Council On School, 2016). Another repetitive activity should be involving parents in the important health-promoting task. Research shows that what school nurses do is not always known (Maenpaa & Astedt-Kurki, 2008). By educating and informing parents, the success rate in reaching adolescents with primary prevention of HPV should increase greatly. It is likely that adolescents will be HPV-vaccinated if the parents have a favorable perception of the HPV vaccination, and school nurses could play a crucial role in this important task. This would be in line with their role as school nurses, as they are trusted both by adolescents and parents (Gottvall et al., 2013; Grandahl et al., 2014; Grandahl et al., 2019).

The participating school nurses were in favor of delivering the educational intervention, pointing out the importance of vaccinations for all adolescents, that is, pan gender vaccination. They thought it was important to reach both girls and boys and emphasized the need for equal health and equity in HPV prevention. Still, it was deemed more difficult to get boys interested as they thought the vaccine had nothing to do with them. The vaccine was initially promoted as a vaccine against cervical cancer, which might be a barrier for parents to accept the vaccine for their son. At the time of the intervention, only girls were offered the vaccine free of charge. This has changed since then, and all adolescents are now included in the national vaccination program, thus increasing the conditions of offering equal care. From the fall of 2020, boys are also offered this vaccine free of charge through the school healthcare program in Sweden. Previous studies indicate that both parents and adolescent boys are in favor of vaccinating against HPV (Gottvall et al., 2017; Grandahl et al., 2019). It was also emphasized that any written material must be inclusive and preferably translated into several languages, given the high number of adolescents in Sweden coming from different countries. Considering the prevalence of HPV-infected women and men, it is hard to comprehend why boys were left out. Surely, it is costly to implement a new vaccination nationally, but the effect of reducing HPV and its possible consequences should be even more cost-effective. Apart from a health economic point of view, individual suffering could also have been reduced, given the high number of women who are diagnosed with HPV-related cervical cancer annually.

School nurses have a key role in the success of HPV vaccination programs, and especially in informing parents and children about the virus and the HPV vaccination (Gottvall et al., 2013; Grandahl et al., 2014). Moreover, school nurses have been identified as advocates for children's health (Boyce & Holmes, 2012; Rose et al., 2011). Consequently, schools are an adequate arena for the prevention of HPV, reaching a wide range of adolescents regardless of sex, sociodemographic status, or cultural background, in line with the UN Convention on the Rights of the Child.

The school nurses in our study expressed that they were flexible in how they held the health consultations with adolescents, in order for the discussion to suit individual needs. The nurses had high ambitions about their work and pointed our personal responsibility as a driving force for achieving high quality. This indicates that the nurses have a strong perception about their role as opinion influencer for the vaccine, which has previously been found to predict positive attitudes regarding the HPV vaccine (Rosen et al., 2016, 2017). It is quite possible that this intervention has increased the nurses’ perception of themselves as opinion influencers as well as improved their knowledge and self-efficacy to provide the HPV vaccine education, which is all factors that could influence school nurses in providing HPV education to adolescents in school and their parents (Rosen et al., 2017, 2019). School nurses have an important role in reducing the prevalence of HPV-related cancers. Increased HPV vaccination rates might decrease individual suffering and, in the end, save lives. The end goal, to eradicate HPV-related cancer, is within reach.

Unanswered questions: Even though the participants expressed positive and encouraging experiences from delivering a complex HPV intervention among adolescents, there are some unanswered questions. In Sweden, approximately 25% of all adolescents have a foreign origin. This means that the material used in the intervention needs to be translated into different languages. It remains to be seen if the intervention works equally well when delivered in other languages. Another unanswered question is how information about HPV can be delivered in other languages even though written material is available. Given the sensitivity of the topic, cultural sensitivity will be a crucial part both in written material and among any healthcare professionals providing the information, pointing out yet another unanswered question: should HPV-related information be adjusted culturally and if so, in what way?

The participating school nurses emphasized the importance of reaching parents with information about HPV. As caregivers must consent to vaccination, it is unknown whether HPV-intervention targeted to adolescents is enough to raise and maintain a high level of HPV-vaccinations. Whether the informational material used in this intervention could be used as part of involving parents and raising awareness about HPV is still to be tested. The participants were positive to HPV-related health promotion and suggested this would be in line with their role as school health nurses. They wished to deliver pan gender vaccinations since this is a way to reduce social inequalities. Furthermore, they pointed out that it would only be possible by having a close cooperation with teachers, principals, and parents. The leaflet was well structured and enabled individually adapted information and health care for the adolescents. However, nurses were concerned that a vast amount of health information has to be covered within current health consultant appointments and requested increased resources in order to meet the growing need for health promotion among adolescents.

School health is an ideal arena for providing adequate information about HPV and promoting prevention. The intervention targeted adolescents who are aged 16, during a time in life when many become sexually active. Since HPV is also a major cause of cancer in the head and neck, anus, vulva, vagina, and penis (Chaturvedi et al., 2011; Du et al., 2012), we believe it is imperative that school nurses continue to provide adequate information about HPV, especially about the link between HPV and sexual behavior.

### Limitations and Suggestions for Future Studies

The included school nurses had experience in the topic at hand and represented various schools situated in different socioeconomic and geographical areas. This resulted in having a wide perspective on the subject. As discussed by Malterud (Malterud et al., 2015), information power indicates that the more information the sample holds, relevant for the actual study, the lower the number of participants needed. The participating school nurses were in favor of the intervention even though it was time-consuming. These nurses might have a more favorable perception about informing about HPV and HPV vaccination compared to school nurses not participating in the educational intervention (Grandahl et al., 2016). The intervention shows promising results in increasing vaccination rates among girls. However, we do not know if the intervention increases vaccination rates among boys. Neither do we know whether they might be more inclined to participate. Further studies are needed. There is an urgent need for interventions developed together with the stakeholders, that is, health care providers, parents, and the children themselves, both boys and girls.

The criteria for assessing the quality and trustworthiness of the conducted study, as described by Guba and Lincoln (Guba & Lincoln, 1989), were: credibility, dependability, conformability, and transferability. Furthermore, as in all qualitative studies, the aim is to gain a deeper understanding of the informants’ thoughts on the subject, not to generalize. Thus, the results may be transferable to similar settings.

In the present study, we wanted to explore the participants’ experiences and views without a predetermined theory, an inductive approach not a deductive approach. However, we believe this is an interesting thought and maybe a theory can be developed over time using several studies in this field. Moreover, we chose focus groups since they are used with the intention to better understand people's views on a subject. A focus group creates a nonjudgmental environment and can encourage participants to share their views without the need for a consensus.

## Implications for School Nursing Practice

School nurses in this study were in favor of delivering the intervention. It suits their work and health-promoting assignment well and can easily be adapted into the current curriculum. However, they pointed out the importance of having written material in different languages to share with adolescents and their parents in order to promote pan gender vaccinations and equal health among adolescents.

## Supplemental Material

sj-docx-1-jsn-10.1177_10598405211046174 - Supplemental material for School Nurses’ Perceptions and Experiences of Delivering a School-Based Intervention to Improve Primary Prevention of Human Papillomavirus Among Adolescents—A Focus Group Study Following a Randomized Controlled Trial Supplemental material, sj-docx-1-jsn-10.1177_10598405211046174 for School Nurses’ Perceptions and Experiences of Delivering a School-Based Intervention to Improve Primary Prevention of Human Papillomavirus Among Adolescents—A Focus Group Study Following a Randomized Controlled Trial by Magdalena Mattebo, Maria Gottvall and Maria Grandahl in The Journal of School Nursing
